# Metabolic profiling of presymptomatic Huntington’s disease sheep reveals novel biomarkers

**DOI:** 10.1038/srep43030

**Published:** 2017-02-22

**Authors:** Debra J. Skene, Benita Middleton, Cara K. Fraser, Jeroen L. A. Pennings, Timothy R. Kuchel, Skye R. Rudiger, C. Simon Bawden, A. Jennifer Morton

**Affiliations:** 1 Chronobiology, Faculty of Health and Medical Sciences, University of Surrey, Guildford GU2 7XH, United Kingdom; 2 Preclinical, Imaging & Research Laboratories (PIRL), SAHMRI, Gilles Plains, Adelaide, Australia; 3 National Institute for Public Health and the Environment (RIVM), 3720 BA Bilthoven, The Netherlands; 4 South Australian Research and Development Institute, Roseworthy, South Australia; 5 Department of Physiology, Development and Neuroscience, University of Cambridge, Downing Street, Cambridge CB2 3DY, United Kingdom

## Abstract

The pronounced cachexia (unexplained wasting) seen in Huntington’s disease (HD) patients suggests that metabolic dysregulation plays a role in HD pathogenesis, although evidence of metabolic abnormalities in HD patients is inconsistent. We performed metabolic profiling of plasma from presymptomatic HD transgenic and control sheep. Metabolites were quantified in sequential plasma samples taken over a 25 h period using a targeted LC/MS metabolomics approach. Significant changes with respect to genotype were observed in 89/130 identified metabolites, including sphingolipids, biogenic amines, amino acids and urea. Citrulline and arginine increased significantly in HD compared to control sheep. Ten other amino acids decreased in presymptomatic HD sheep, including branched chain amino acids (isoleucine, leucine and valine) that have been identified previously as potential biomarkers of HD. Significant increases in urea, arginine, citrulline, asymmetric and symmetric dimethylarginine, alongside decreases in sphingolipids, indicate that both the urea cycle and nitric oxide pathways are dysregulated at early stages in HD. Logistic prediction modelling identified a set of 8 biomarkers that can identify 80% of the presymptomatic HD sheep as transgenic, with 90% confidence. This level of sensitivity, using minimally invasive methods, offers novel opportunities for monitoring disease progression in HD patients.

Huntington’s disease (HD) is a genetic neurodegenerative disorder caused by an unstable CAG repeat mutation in *HTT*^1^. It is invariably fatal and there are no treatments targetting the molecular cause of the disease. Although HD is diagnosed by the presence of chorea, it is well recognised that HD is not simply a motor disorder. Psychiatric disturbance, cognitive decline and sleep/circadian abnormalities all contribute to the insidious decline of HD patients. Furthermore, while progressive neurodegeneration of the brain is the best characterised pathological hallmark of HD, recent studies have also identified peripheral pathologies as potentially important components of HD pathogenesis. These include cardiomyopathy (for references, see^2^,^3^) and the pronounced skeletal muscle wasting known as cachexia (Refs 4, 5, 6, 7; for other references see^3^,^8^). Indeed, cachexia is one of the best recognised signs of HD, and appears to be an inevitable sign in HD patients at end stages of disease. Numerous studies have shown that weight loss in HD is not secondary to poor nutrition, because HD patients have normal or even higher calorific intake than control subjects (for references, see^1^). Skeletal muscle dysfunction caused by cachexia and loss of motor control causes motor symptoms including dysarthria (inability to talk) and dysphagia (swallowing difficulties). Dysphagia causes the aspiration pneumonia that is one of the major causes of morbidity in HD patients^9^,^10^.

The dual presence of chorea and cachexia in HD stimulated the first studies of metabolism in HD in the 1960s. Initially it was thought that the chorea caused the cachexia, by using excess energy. It is now known that patients with a greater number of CAG repeats exhibit a more rapid loss of weight^11^, and that cachexia is accompanied by changes in gene expression and metabolism that are likely to affect whole-body metabolism and function. Interestingly, cachexia is also a prominent feature of other important diseases such as Alzheimer’s disease and cancer, as well as ageing. The mechanism underlying cachexia in all diseases is an increased breakdown of muscle protein, which coupled with reduced protein synthesis, leads to overall muscle loss^8^,^12^,^13^,^14^. These pathways are likely to be disrupted in HD, but the precise mechanism and time course of their disruption is unknown.

Until recently, results from metabolic studies in HD have been very variable and for the most part, insubstantial. In early studies, particular attention was paid to lipid and protein metabolites, and although some changes were seen, none explained the remarkable wasting of HD patients. Later, direct study of mitochondrial function from HD patients and HD mice was undertaken, as have been large-scale metabolomics studies^15^,^16^,^17^. Mitochondrial abnormalities have been implicated in metabolic changes, with reduced mitochondrial function found in both HD patient lymphoblasts and HD mouse models^18^,^19^,^20^. A number of untargeted metabolomic studies using both humans^16^,^21^,^22^ and HD rodent models^23^,^24^ have given interesting results, hinting at, but not always revealing, substantial changes in metabolic pathways in HD. Others have found neither changes in energy metabolism^17^ nor carbohydrate, protein or lipid metabolism markers that can differentiate between healthy controls, premanifest and stage II/III HD subjects^25^. Most recently, however, Cheng *et al*.^26^ found some changes in metabolic profiles of HD patient plasma, Graham *et al*.^27^ used NMR spectroscopy to identify a metabolic signature of HD, and Patassini *et al*.^28^ revealed significant metabolic changes in post mortem human brain. These latest studies strongly support the idea that metabolism is deranged in HD, although none show the same changes, and some findings conflict with each other^27^,^28^. For example, Patassini *et al*. showed that brain urea levels increased significantly, Graham *et al*. found that they decreased. The differences between each study exemplify the difficulty in controlling innate metabolic variation in humans. Nevertheless, there is a consistent theme of dysregulated metabolism in all of these studies, particularly with respect to mitochondrial function, nitrogen metabolism and lipid metabolism.

Part of the problem with inconsistency between studies lies in the fact that there are considerable challenges associated with measuring metabolism in humans. Diet, lighting conditions, sleep/wake status and time of day of sampling all have a profound effect on metabolic profiling^29^. Diet and lighting conditions can be controlled, with difficulty in patients, but are more easily managed in animal models. Endogenous circadian variation, however, is more problematic for metabolic studies, since this requires highly controlled laboratory conditions -the so-called constant routine protocol - to minimise the effect of exogenous factors on circadian rhythmicity^30^. There is clear evidence that circadian rhythms are disrupted in HD patients^31^,^32^, mice^31^,^33^,^34^,^35^ and sheep^33^, and that circadian regulation of hepatic metabolites in HD mice is abnormal^36^. It is thus possible that differences in metabolites, particularly those that are circadian-regulated, may be missed or masked in samples from subjects with circadian defects, if the samples are not collected ‘around-the-clock’. The ideal sampling regime is to take samples at least 2 hourly from subjects with controlled dietary intake, in dim light over a 24 hour time period. However, such sampling regimes are difficult and expensive to conduct in HD patients, and near impossible in mice. For this reason, we used a transgenic sheep model of HD, since the feeding and housing conditions of sheep can be well controlled, and they are large and robust enough to tolerate multiple blood sampling over the course of ~24 hours.

Metabolomics is the profiling of small-molecule metabolites (<1 kDa). It offers promise for studying not only homeostatic regulation but also system perturbations that can be caused by genetic changes, microbes and disease. Metabolomics has an advantage over other “omics” technologies, in that it assesses directly the metabolic changes in an organism, providing a better representation of functional phenotype than changes at the gene, transcript, and protein levels^37^. Metabolic profiling thus has the potential to identify novel biomarkers that could be used as diagnostic tools for disease progression or response to treatment. Key metabolomics methodologies include nuclear magnetic resonance (NMR), and mass spectrometry (MS)-based technologies (namely liquid chromatography (LC)/MS and gas chromatography (GC)/MS, with LC/MS being the most sensitive). Targeted metabolomics methods using stable isotope-labelled standards have the advantage over untargeted metabolomics methods of allowing the researcher to quantify select classes of metabolites, allowing changes in metabolic function to be characterised.

Identification of metabolic biomarkers and the metabolic pathways associated with HD progression will aid understanding of the pathophysiological mechanisms of the disease, and also spur development of effective therapies by providing sensitive measures of disease progression (for review see ref. 38). With therapeutic trials of novel therapies commencing apace in HD, the need for a robust biomarker, has never been greater. The difficulties in studying both people (with their difficult-to-control lifestyles that modulate metabolism) and mice (with their fast rodent metabolism and small blood volume for repeated sampling) would be greatly alleviated if it were possible to use a large animal model with metabolism similar to that of humans that could be tested in a controlled environment. The recently developed ovine model of HD (OVT73) offers such an opportunity^39^. This sheep carries a CAG repeat expansion of 73 in a full-length human cDNA transgene. Lack of overt behavioural abnormalities and absence of structural brain changes^31^, combined with only subtle neuropathology^40^ and minor sex-dependent post mortem changes in cerebellum and liver metabolism^41^ suggest that, at 5 years of age, this line of sheep is still at a presymptomatic stage of HD. Here we used a targeted LC/MS based metabolomics strategy to assess biochemical alterations and identify potential biomarkers in presymptomatic HD sheep (hereafter called ‘HD sheep’).

## Results

**Figure 1:**
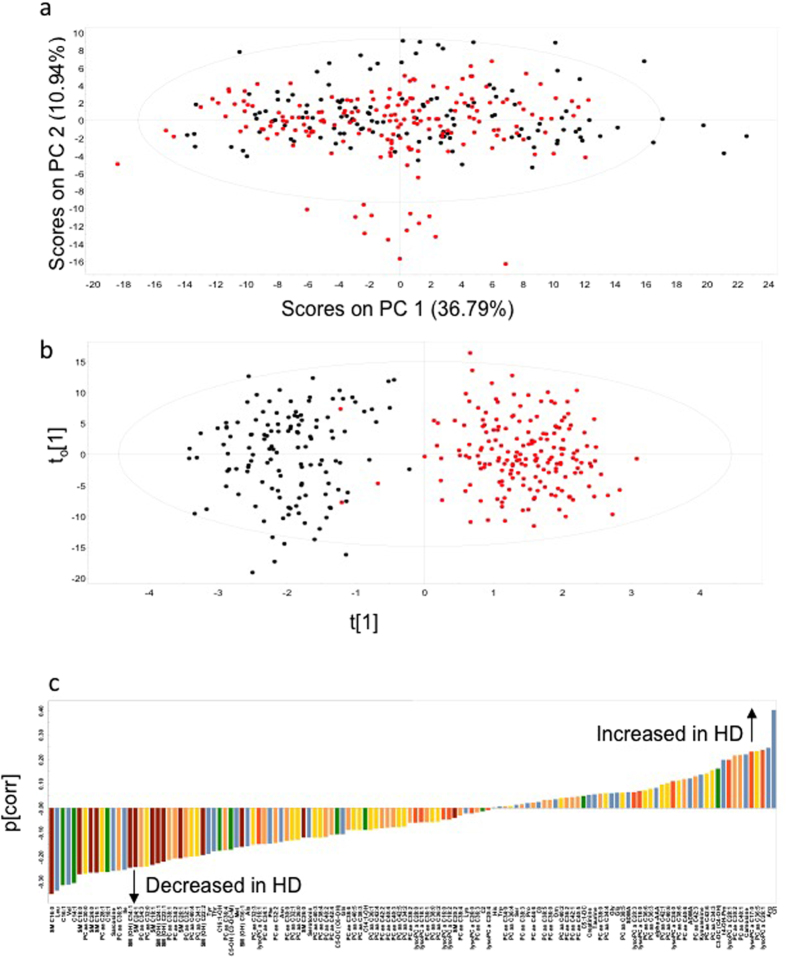
Multivariate analysis of targeted metabolomics data. (**a**) Principal component analysis (PCA) of all the data (n = 24 sheep, 10 control, 14 HD; n = 130 metabolites; n = 13 time points). Samples are coloured by genotype (black, control; red, HD); (**b**) OPLS-DA model showing separation by genotype. (**c**) OPLS-DA loading plot of control vs HD. Negative p(corr) values represent decreased and positive p(corr) values represent increased metabolite concentrations in HD compared to control sheep. The metabolite bars are colour coded according to metabolite class as follows: amino acids and biogenic amines (blue); acylcarnitines (green); lysophosphatidylcholine acyl (lyso PC a) (dark orange); phosphatidylcholine diacyl (PC aa) (yellow); phosphatidylcholine acyl-akyl (PC ae) (light orange); sphingolipids (SM) (brown). For further details on metablolites, see text and Suppl. Table 2.

Targeted LC/MS metabolomics was used to examine the effect of genotype (HD compared to control sheep) on plasma metabolite concentrations. Principal component analysis (PCA) of all the samples (n = 24 sheep; 10 controls, 14 HD), time points (n = 13) and metabolites (n = 130) was performed, the PC1 versus PC2 scores matrix is presented in Fig. 1a. Abbreviations used for metabolites are shown in full in Table 1. Metabolites (ordered left to right in the figure) are 1.SM C16:0; 2. Leucine; 3. Hexadecenoylcarnitine; 4. Valine; 5. Tetradecenoylcarnitine; 6. SM C18:0; 7. PC aa C30:0; 8. SM C24:0; 9. SM C16:1; 10. PC aa C28:1; 11. Octadecenoylcarnitine; 12. Sarcosine; 13. PC ae C36:5; 14. Isoleucine; 15. SM (OH) C14:1; 16. SM C24:1; 17. PC ae C34:3; 18. PC aa C32:0; 19. SM C18:1; 20. SM (OH) C24:1; 21. SM (OH) C22:1; 22. PC ae C38:1; 23. PC ae C34:2; 24. SM C26:1; 25. PC ae C32:1; 26. PC aa C40:4; 27. PC aa C34:1; 28. SM (OH) C22:2; 29. Tyrosine; 30. Threonine; 31. Hydroxyhexadecenoylcarnitine; 32. PC ae C36:4; 33. Hydroxyvalerylcarnitine; 34. Methionine; 35. SM (OH) C16:1; 36. Alanine; 37. PC aa C32:3; 38. lysoPC a C16:1; 39. PC ae C34:1; 40. Phenylalanine; 41. PC ae C32:2; 42. Asparagine; 43. PC ae C30:0; 44. PC aa C32:1; 45. PC aa C36:0; 46. SM C26:0; 47. Serotonin; 48. PC aa C40:3; 49. PC aa C38:4; 50. PC ae C40:2; 51. PC ae C42:4; 52. Glutarylcarnitine; 53. Glutamine; 54. PC ae C38:6; 55. PC aa C40:5; 56. PC aa C38:3; 57. Hydroxytetradecenoylcarnitine; 58. PC aa C36:1; 59. PC aa C42:4; 60. PC ae C42:2; 61. PC ae C40:4; 62. PC ae C40:3; 63. PC aa C42:5; 64. PC aa C34:2; 65. PC ae C38:2; 66. lysoPC a C28:0; 67. lysoPC a C18:1; 68. PC ae C36:1; 69. PC aa C38:0; 70. PC aa C36:2; 71. lysoPC a C16:0; 72. lysoPC a C18:2; 73. SM C20:2; 74. PC ae C38:4; 75. Lysine; 76. lysoPC a C26:0; 77. PC ae C36:3; 78. Acetylcarnitine; 79. lysoPC a C20:4; 80. Histidine; 81. Tryptophan; 82. PC ae C34:0; 83. PC aa C36:4; 84. Serine; 85. PC ae C38:3; 86. Proline; 87.PC ae C44:6; 88. Carnitine; 89. PC ae C38:5; 90. PC ae C36:0; 91. Ornithine; 92. PC aa C40:2; 93. PC ae C36:2; 94. PC ae C42:1; 95. PC ae C40:5; 96. Glutaconylcarnitine; 97. Creatinine; 98. Taurine; 99. PC ae C38:0; 100. PC aa C34:4; 101. Glycine; 102. Glutamate; 103. PC aa C38:5; 104. Symmetric dimethylarginine; 105. lysoPC a C20:3; 106. lysoPC a C18:0; 107. PC aa C36:6; 108. PC aa C36:3; 109. alpha-Aminodipic acid; 110. PC aa C42:1; 111. PC aa C40:6; 112. lysoPC a C24:0; 113. PC aa C38:6; 114. PC ae C40:6; 115. Asymmetric dimethylarginine; 116. PC ae C42:3; 117 Kynurenine; 118. PC aa C42:6; 119. PC aa C34:3; 120. Malonylhydroxybutyrylcarnitine; 121. Trans-4-hydroxyproline; 122. lysoPC a C28:1; 123. PC ae C30:2; 124. PC ae C40:1; 125. Carnosine; 126. lysoPC a C17:0; 127. PC aa C36:5; 128. lysoPC a C26:1; 129. Arginine; 130. Citrulline. No separation of genotype was evident in this unsupervised PCA. However, orthogonal partial least squares discriminant analysis (OPLS-DA) models, validated by permutation analysis, showed good separation between the control and HD sheep (Q^2^ (cumulative) = 0.790, total amount of variance explained in the x matrix (R^2^X) (cumulative) = 0.687; total amount of variance explained in the y matrix (R^2^Y) (cumulative) = 0.863; Fig. 1b). The p(corr) loading plot for the OPLS-DA model is shown in Fig. 1c and the p(corr) values for each metabolite are presented in Supplementary Table 1. Increased concentrations of citrulline, arginine, carnosine, t4-hydroxy-proline (t4-OH-proline), kynurenine, asymmetric dimethylarginine (ADMA), alpha-aminoadipic acid (alpha-AAA) and symmetric dimethylarginine (SDMA) and decreased concentrations of amino acids (particularly the branched chain amino acids), acylcarnitines, sarcosine and sphingolipids were observed in the HD sheep. There was a significant correlation between the p(corr) values and genotype False Discovery Rate (FDR) values obtained from the ANOVA analyses of the metabolite concentrations. The top 20 metabolites at the extreme ends of the loading plot (Fig. 1c) also showed the most significant effect of genotype in the ANOVA analyses of the metabolite concentrations (Table 1). In total, 89 of the 130 metabolites (68%) changed significantly with respect to genotype. Of these, 25 metabolites showed increased and 64 metabolites showed decreased concentrations in HD sheep compared to controls (FDR < 0.05; Table 1). A heat map combined with hierarchical clustering of these metabolite profiles across the 24 h period is shown in Supplementary Figure S1.

### Amino acids

**Figure 2:**
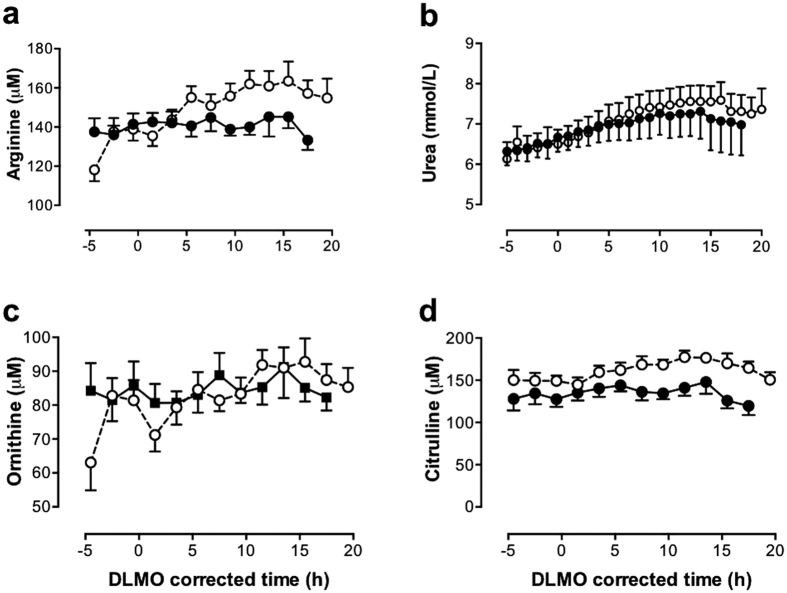
Twenty four-hour profiles of urea cycle metabolites. (**a**) Arginine, (**b**) urea, (**c**) ornithine, (**d**) citrulline. Mean (±SEM) plasma levels in control (◾, solid line) and transgenic HD (○, dashed line) sheep are presented, corrected for circadian phase using each sheep’s dim light melatonin onset (DLMO), annotated as zero (=20.9 ± 0.9 h (mean ± SEM) ≈21.00 h). Significantly increased arginine, urea and citrulline concentrations were observed in the HD sheep compared to the controls (FDR < 0.05).

**Figure 3:**
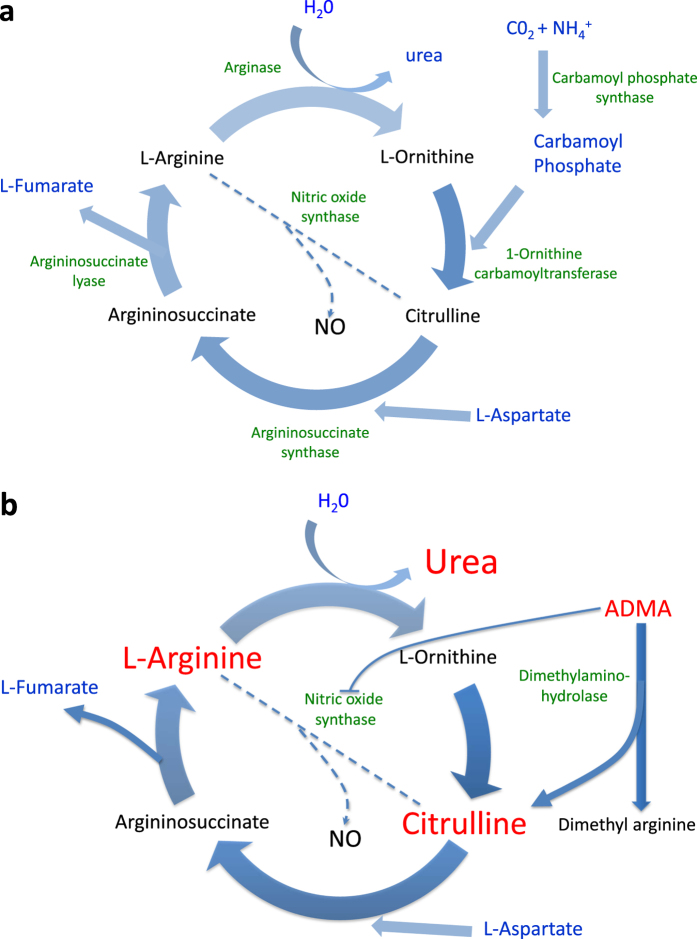
The urea cycle and nitric oxide (NO) pathway in health and disease. (**a**) The major constituent parts of the metabolic pathway that comprises the urea cycle and NO pathway. (**b**) Changes in metabolites of the urea cycle and NO pathway in HD sheep. Significant increases are shown in red font. Abbeviations: NO = nitric oxide, ADMA = asymmetric dimethylarginine

**Figure 4:**
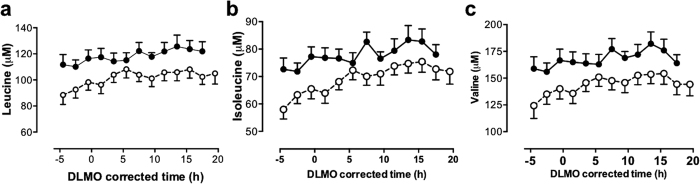
Comparison of 24-h profiles of branched chain amino acids in normal and transgenic HD sheep. (**a**) Leucine, (**b**) isoleucine, (**c**) valine. Data are mean (±SEM) plasma levels in control (◾, solid line) and HD (○, dashed line) sheep. Data are corrected for circadian phase using each sheep’s dim light melatonin onset (DLMO) annotated as zero (20.9 ± 0.9 h (mean ± SEM) ≈21.00 h). Significantly reduced branched chain amino acids were observed in the HD sheep compared to controls (FDR < 0.05).

Of the 20 amino acids quantified, only citrulline and arginine had significantly increased levels in HD compared to control sheep (Fig. 2). Indeed, of all 130 metabolites quantified, citrulline showed the most marked change (FDR < 1.0E-23) in the HD sheep. Citrulline and arginine are both part of the urea cycle (Fig. 3). However ornithine, also part of the urea cycle, showed no significant differences between the genotypes (Fig. 2). Of the 20 amino acids measured, 10 had significantly reduced concentrations in the HD sheep, with the branched chain amino acids (valine, leucine, isoleucine) showing the most pronounced effect of genotype followed by threonine, tyrosine, methionine, alanine, asparagine, phenylalanine and glutamine (Table 1, Fig. 4).

The ratios of Cit/Arg, Cit/Orn and Orn/Arg were calculated as indicators of nitric oxide synthase (NOS), ornithine carbamoylphosphate transferase and arginase activity, respectively. All of the ratios were significantly different (FDR < 1.0E-12) between the HD and control sheep, with higher Cit/Arg and Cit/Orn ratios and a lower Orn/Arg ratio in the HD sheep.

### Biogenic amines

**Figure 5:**
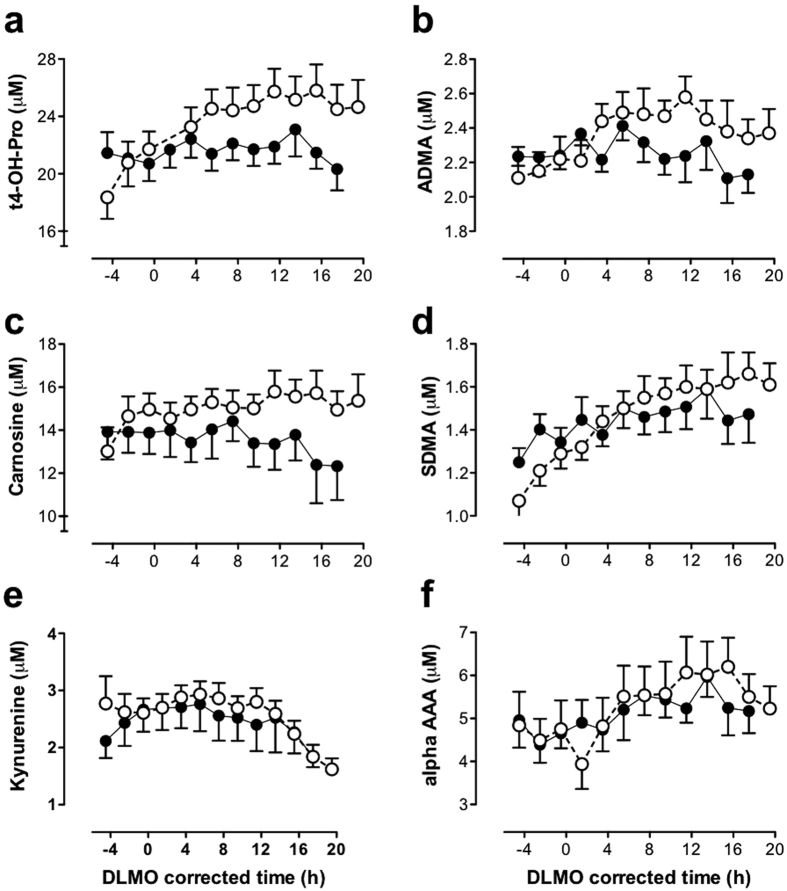
Comparison of 24-h profiles of biogenic amines in normal and transgenic HD sheep. (**a**) Trans-4-hydroxyproline (t4-OH-proline), (**b**) carnosine, (**c**) kynurenine, (**d**) asymmetric dimethylarginine (ADMA), (**e**) symmetric dimethylarginine (SDMA) and (**f**) alpha-aminoadipic acid (alpha-AAA). Mean (±SEM) plasma levels in control (◾, solid line) and HD (○, dashed line) sheep are presented, corrected for circadian phase using each sheep’s dim light melatonin onset (DLMO), annotated as zero (=20.9 ± 0.9 h (mean ± SEM) ≈21.00 h). All these biogenic amines were significantly increased in HD sheep compared to controls (FDR < 0.05).

Seven of the 9 quantified biogenic amines changed significantly with respect to genotype. Six of these increased significantly in HD sheep. These were trans-4-hydroxyproline (t4-OH-proline) >carnosine >kynurenine >ADMA >SDMA >alpha-AAA (Fig. 5). By contrast, only sarcosine showed significantly reduced levels (FDR < 1.0E-19) in the HD sheep.

### Acylcarnitines

Most of the measured acylcarnitines (8 of 11) were significantly altered by genotype, 6 showing reduced concentrations and two (hydroxybutyrylcarnitine (C3-DC(C4-OH)) >glutaconylcarnitine (C5-1-DC)) showing increased concentrations (Table 1).

### Sphingolipids

**Figure 6:**
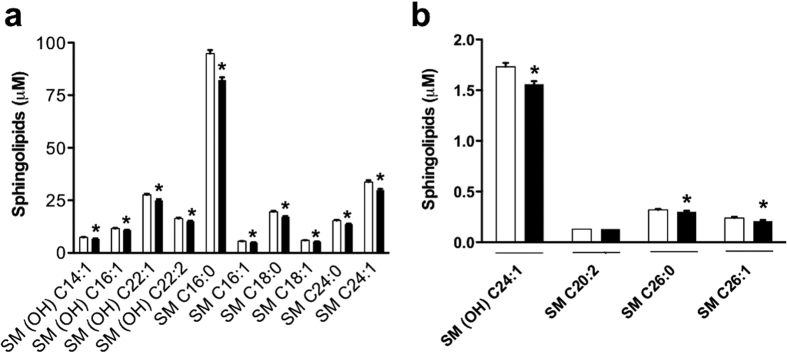
Comparison of plasma concentrations of 15 sphingolipids in normal and transgenic HD sheep. Panels show sphingolipids with concentrations between 2 and 100 μM (**a**) or up to 2 μM (**b**). Data are 24 h mean values (±SEM) derived from all the time points. Most (14/15) of the sphingolipids were significantly reduced in HD sheep compared to controls (* FDR < 0.05).

All, but one (SM C20:2), of the 14 sphingolipids (93%) quantified were significantly reduced in the HD sheep compared to the controls (Table 1). These 13 sphingolipids included both hydroxylated (n = 5) and non-hydroxylated (n = 8) ceramide phosphocholines (sphingomyelins). Figure 6 presents the 24 h mean data of each of the 15 sphingolipids in the HD and control sheep.

### Glycerophospholipids

Of 75 quantified glycerophospholipids (n = 62 phospholipids; n = 13 lysophosphatidylcholines), 44 phospholipids (71%) and 5 lysophosphatidylcholines (38%) were significantly altered in the HD sheep (Table 1). There was no consistent pattern to the direction of change with some showing markedly reduced levels (e.g. PC ae C38:1; PC aa C34:1; PC aa C30:0; PC aa C28:1; PC aa C40:4) and some showing elevated levels (e.g. PC aa C36:5; lysoPC a C17:0; PC ae C30:2) in the HD sheep (Table 1).

### Urea

The hourly plasma concentrations of urea nitrogen in the control and HD sheep are presented in Fig. 2. Urea nitrogen levels were significantly higher (FDR < 1.0E-18) in the HD sheep (7.0 ± 0.08 mmol/L) compared to the control sheep (6.8 ± 0.09 mmol/L).

### Quantitative enrichment analysis (QEA)

Quantitative enrichment analysis (QEA) was performed using MetaboAnalyst 3.0 (www.metaboanalyst.ca; ref. 42). The top 5 metabolite pathway-associated metabolite sets were aspartate metabolism; arginine and proline metabolism; valine, leucine and isoleucine (branched chain amino acids) degradation; fatty acid metabolism and urea cycle (Supplementary Figure S2). No pathways, however, were significantly enriched following FDR correction.

### Prediction modelling

**Figure 7:**
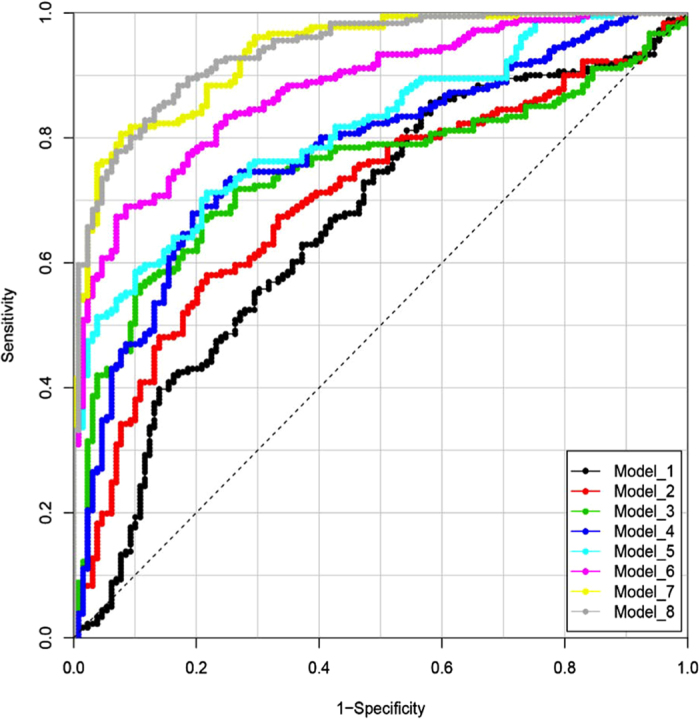
Receiver operating characteristic plots of the 8 prediction models. Models were improved stepwise by including an additional metabolite until no more gain in AUC could be obtained during cross-validation. The metabolites used in each model are shown in Supplementary Table 2.

Using logistic prediction modelling, we developed eight models with increasing performance by adding one metabolite at a time to the existing model until no further improvement was obtained (Fig. 7). The simplest of these was based only on citrulline (model_1, AUC = 0.664, sensitivity = 17.7%). Stepwise improvement combined with leave-one-sheep-out cross-validation resulted in a final model (model 8) based on eight markers (citrulline, valine, PC aa C40:4, PC aa C36:5, lysoPC a C17:0, SM (OH) C24:1, threonine, tetradecenoylcarnitine (C14:1)) with an AUC of 0.938. This model allowed for 80.1% sensitivity at 90% specificity (Supplementary Table 2). In the HD sheep citrulline, PC aa C36:5 and lysoPC a C17:0 were significantly increased and valine, PC aa C40:4, SM (OH) C24:1, threonine and tetradecenoylcarnitine (C14:1) were significantly decreased compared to the control sheep.

In order to determine which time point best discriminates the control and HD groups, AUC values for the models were determined for each time point. For the final (best performing) model (model 8), the 05:00 h time point gave the best discrimination between the genotypes (AUC = 0.986). Times 07:00 h and 09:00 h perform as second/third best (AUC = 0.971 for both). For comparison, the AUC for all time points combined is 0.938, so the additional discrimination for these time points is relatively small. When other models were also considered, time points 05:00 h and 07:00 h perform as best and second-best respectively, in the majority of the models. The time point 05:00 h (before dawn) therefore seems the most discriminating between the genotypes.

## Discussion

Using targeted LC/MS based metabolomics we have identified significant metabolic alterations in the HD sheep. The metabolites we identified include metabolites that have been identified previously as being involved in HD pathology (kynurenine, urea) or suggested as biomarkers for HD (branched chain amino acids). Together our data strongly support the idea that a plethora of metabolic changes occur very early (and even presymptomatically) in HD, and that these are likely to contribute deleteriously to its progression.

Marked elevation in the amino acids citrulline and arginine, both components of the urea cycle, were observed in the HD sheep. Our findings corroborate previous studies reporting raised blood citrulline levels in patients with HD and in two mice models (R6/2 and HdhQ150; ref. 43) and are consistent with abnormalities in the urea and NO cycles. A suggested mechanism is that disruption of CCAAT-enhancer-binding proteins (C/EBP) activity by mutant huntingtin^44^ causes suppression of the expression of two key enzymes (argininosuccinic acid synthetase and argininosuccinase acid lyase) of the urea cycle^44^. In addition to being produced in the urea cycle from ornithine and carbamoyl phosphate, citrulline is derived from arginine as a by-product of NO synthesis via NO synthase. The increased arginine observed in the HD sheep may thus contribute to raised citrulline and NO levels (Fig. 3b). Although we could not measure NO levels using the samples we collected for LC/MS analysis, it would be interesting to measure NO levels in any future studies. Raised levels of both arginine and NO have been implicated in HD progression^25^. Dietary supplementation of arginine in HD mice hastens disease progression (demonstrated by increased weight loss and abnormal motor function; ref. 43). Furthermore, in a number of other studies NO and NOS have been linked either directly or indirectly to many aspects of HD pathology, such as oxidative stress^45^, mitochondrial dysfunction^46^,^47^, platelet signalling^48^ and peripheral vasodilatation^49^. The significant increase in the Cit/Arg and Cit/Orn ratios in the HD sheep suggest that abnormal activation of the enzymes nitric oxide synthase (NOS) and ornithine carbamoylphosphate transferase is likely. Until we conducted the analysis, we did not know that the urea cycle or NO system might be perturbed in the HD sheep, and we could not measure NO levels in the plasma retrospectively. The decreased Orn/Arg ratio suggests suppression of arginase (ARG) activity in the HD sheep. Together these data point to abnormalities in the urea cycle and NO cycle, as has been suggested in HD mice^44^,^49^,^50^. Interestingly, a low protein diet (17%) restores urea cycle activity and ameliorates symptoms in HD model mice^43^. In support of possible NO disturbances in HD, the HD sheep also had significantly raised levels of ADMA and SDMA compared to control sheep. ADMA is an endogenous inhibitor of NOS (inhibiting all 3 isoforms of NOS; ref. 51; Fig. 3b). SDMA, the structural isomer of ADMA, competes with the arginine transporter, and regulates NOS by limiting amounts of ADMA^52^. ADMA, but not SDMA, is hydrolysed by dimethylarginine dimethylaminohydrolase (DDAH) to form dimethylamine (DMA) and citrulline. Thus an increase in ADMA could cause/contribute to an increase in citrulline. Raised plasma levels of ADMA and SDMA are associated with a range of conditions, including ageing, cardiovascular disease, hypercholesterolaemia and insulin resistance/hyperglycaemia (for review, see ref. 53). Finally, there is evidence for abnormal processing of arginine playing a role in HD^54^.

A prominent finding in the current study was the marked decrease in plasma sphingolipids in HD sheep compared to controls. Progressive neurodegeneration in HD involves loss of both grey and white matter volume and myelin breakdown. This may correlate with reduced sphingolipids in the circulation. Sphingolipids are involved in the structure and function of cell membranes in the brain^55^,^56^ and are associated with a plethora of biological functions^57^. Dysfunction in the synthesis and/or breakdown of sphingolipids would be expected to have a major impact on neuronal function, neurotransmitter receptors, synaptic transmission and cellular signalling, in addition to the myelination/oligodendrocyte deficits. There is accumulating evidence that NO and sphingolipids interact in reciprocal pathways, leading to regulation of activity and expression of enzymes involved in signalling events from both pathways (for review, see ref. 58). Using an untargeted metabolomics approach, sphingolipids have also been identified recently as dysregulated in an HD cell line model^59^.

Notwithstanding the fact that it may be difficult to link brain processes with plasma biomarkers, there are numerous studies to suggest that metabolic changes in the periphery reflect a disease signature. Peripheral sphingolipids have been reported to be predictors of cognitive decline, low sphingomyelin levels being associated with a faster rate of cognitive decline^60^,^61^. In an effort to understand the relationship between central and peripheral processes, metabolic profiling has been performed on both CSF and plasma taken from the same individuals (controls, mild cognitive impairment and Alzheimer’s disease^62^). These authors showed that approximately 30% of the metabolic pathways altered in the CSF of the study groups were also altered in the plasma, *inter alia* sphingolipid transport pathways were significantly affected in both CSF and plasma, validating plasma as a useful biofluid for neurodegenerative disease research. However, even if these long chain sphingolipids do not cross the blood brain barrier and there is no direct correlation between CSF and peripheral levels, it does not mean that plasma sphingolipids will not be useful as biomarkers in HD prognosis. As second messengers there are several mechanisms by which sphingolipids could directly or indirectly affect HD disease progression, as has been shown for Alzheimer’s disease^63^,^64^. These findings, combined with the present study, suggest that sphingolipids may be interesting general markers of neurodegeneration. It would be particularly interesting if this were the case, since there is currently no structural^31^ and only subtle histological^40^ evidence to show neurodegeneration in these HD sheep. If plasma sphingolipids herald incipient neurodegeneration, it may be useful as a predictive biomarker. This would be a particularly useful aspect of the metabolome on which to focus in the future.

The present findings support our previous observations of dysregulation of a number of nitrogen metabolising genes (*Ahcy, Ass1* and *Arg1*) that might contribute to HD symptoms^36^. Dysregulation of *Ass1* and *Arg1,* key components of the urea cycle, could lead to the accumulation of toxic metabolites that could exacerbate HD neuropathology^65^,^66^. Interestingly, urea cycle abnormalities characterized by hyperammonemia, high blood citrulline, and suppression of urea cycle enzymes have already been shown in two mouse models of HD as well as in HD patients^44^,^67^. Finally, elevated levels of urea have been found in post-mortem HD brain^28^.

Ten other amino acids measured were significantly reduced in the HD sheep. Of particular note were the proteinogenic branched chain amino acids (leucine, isoleucine and valine). These have previously been identified as possible biomarkers for HD^68^,^69^. That study, using a large cohort of patients with HD at different stages of the disease, showed a significant reduction of plasma branched chain amino acids in HD, which correlated with weight loss and disease progression. Leucine was of particular interest because it was significantly reduced in patients at an early stage of the disease, as well as in presymptomatic individuals. Leucine is a well-known activator of mTor (mammalian target of rapamycin2), which regulates protein synthesis and whose inhibition results in increased autophagic proteolysis. It is particularly notable that the branched chain amino acids were decreased in the HD sheep that are all at least 3 years from disease onset. (This study was done in 5 year old sheep that are now 7-8 years old and still showing no symptoms; unpublished data; AJM). This supports the suggestion that branched chain amino acids may be a useful biomarker for HD. Consistent with our findings, another group suggested that asparagine and serine (revealed in all of our statistical analyses) could be considered as potential plasma HD biomarkers^70^.

Our decision to use a targeted rather than an untargeted metabolomics approach was pragmatic, based on the large number of samples generated by round-the-clock sampling (13 samples for each animal, n = 24 sheep) and the cost/time involved, which is much greater for untargeted than targeted metabolomics analysis. While untargeted metabolomics analysis may generate additional metabolites, without the intensive workup necessary to identify these metabolites according to the Metabolomics Standards Initiative (www.metabolomics.msi.org; ref. 71), they would only remain as ‘features’ or ‘putative metabolites’ with mass-to-charge ratio (m/z) information. This is less useful than the identified metabolite that is available with the targeted approach. By analysing our samples using targeted metabolomics analysis we obtain absolute concentrations of the metabolites (as opposed to relative expression in untargeted analysis). Although the total number of metabolites measured using targeted metabolomics analysis is relatively small, the method is nevertheless extremely useful for understanding changes in metabolic function in different physiological and pathological states. In addition, the use of a fully validated method that is selective and reproducible with low CV gives confidence that the metabolites identified by this method are real and that the data generated can be replicated in other facilities.

Taking advantage of being able to measure metabolites from 5 different classes and using logistic prediction modelling, an optimised marker panel containing 8 biomarkers was identified in our metabolite panel. These metabolites (citrulline, valine, PC aa C40:4, PC aa C36:5, lysoPC a C17:0, SM (OH) C24:1, threonine, C14:1) come from 4 of the 5 metabolite classes quantified. With this panel of 8 metabolites, we were able to identify 80% of the HD sheep as transgenic, with 90% confidence. Identifying 8 biomarkers that can be measured reliably and relatively easily makes this a method that could be adopted by a large number of laboratories and translated to studies in human subjects.

As well as providing evidence for the presence of metabolic biomarkers of HD, the changes we see strongly validate the use of the OVT73 sheep as a model of HD. The particular value of a sheep model of HD in general, and these sheep in particular, is that they can be monitored longitudinally to determine if/how these metabolic changes progress over time. If so, the metabolic changes may be useful as a state or rate markers that could eventually be used to monitor disease progression. We identified more dysregulated metabolites than have been identified in any other study. This is likely to be because of our sampling regime. There has never been a study using an HD animal model in which around the 24 h clock samples have been taken under well-controlled conditions. It is interesting to note that, while no study in patients shows changes in all of the metabolites we identified, all of the metabolites or metabolic families have been identified previously as being dysregulated in HD. Clearly, the set of metabolites we have identified as potential biomarkers will also need to be measured in HD patients under well-controlled conditions, to confirm the findings from our sheep model. The need for a robust biomarker, however, has never been greater. Currently, clinical outcome measures are used for tracking the progression of HD. Measures such as the unified HD rating scale (UHDRS) are well established and widely used. They are, however, subjective, and prone to rater variability. Furthermore, because they are based on symptomatic assessment, they cannot be used to detect preclinical changes in gene mutation carriers. Much progress has been made in recent years in developing and evaluating biomarkers for HD, most notably in the fields of quantitative clinical measures and cognitive measures, but the development of objective and reliable disease-specific biomarkers that can be rapidly measured become immeasurably important once clinical trials for therapies begin. This is more important now than it has ever been, since several novel therapeutic candidates are in ongoing or planned clinical trials, including the first clinical trial of a gene knockdown therapy using an antisense oligonucleotide therapeutic called Ionis-HTTR_x_ 72.

In summary, the metabolic profiles reveal clear urea cycle and NO system disturbances in presymptomatic HD sheep. A panel of 8 biomarkers that confidently detects disease in presymptomatic HD sheep is proposed. A biomarker that can be used to define the stage or track the progression of disease would be invaluable for measuring therapeutic effects in HD.

## Materials and Methods

### Animals

Twenty four merino rams (*Ovis aries)* were used for this study. HD transgenic (OVT73 line) and control rams were maintained outdoors as a mixed transgenic/normal ram flock in PC1-certified paddocks (PC1 Large Grazing Animal Facilities) at the South Australian Research and Development Institute (SARDI) in accordance with AEC approval #27/13 (Primary Industries and Regions South Australia; PIRSA) and Office of the Gene Technology Regulator (OGTR) approval NLRD-1037/2003. They were born and raised on this site and were 5 years old [at the time of this study]. Up to the time of blood sampling, rams were grazed on pasture *ad libitum*, with feed supplementation during seasonal periods when feed-on-offer was low. For circadian blood sampling, 24 (14 HD and 10 control) rams were transported from SARDI to the Preclinical Imaging and Research Laboratory (PIRL) of the South Australian Health and Medical Research Institute (SAHMRI) at Gilles Plains, South Australia. Animal husbandry and blood sampling was performed in accordance with AEC approvals #07/14 (PIRSA) and SAM93 (SAHMRI). For at least 4 weeks prior to transport the rams were fed a ration of cereal hay and a pea/barley mixture supplement that was continued once the sheep had been relocated to PIRL. The aim was to ensure minimal dietary change throughout the period prior to sampling when sheep were moved from one site to the other.

### Animal husbandry (acclimatisation, feed adaption and fasting)

Sheep were acclimatised at SAHMRI for three weeks following transfer from SARDI (May, 2014). The holding areas had ambient light. Sheep were fed on a diet of feed that included a combination of Oats/Lucerne/Vetch 2013 season hay and Ewe replacement sheep nuts (Laucke Mills). Sheep were transferred into undercover pens for a minimum of one week acclimatisation to human contact. Sheep were transferred to indoor holding on the day prior to cannula insertion. They were housed in individual metabolic crates elevated approximately one metre above the floor.

During indoor acclimatisation the light cycle matched the outdoor light/dark cycle. At the time sampled, this was ~10 h light; 14 h dark (10 L:14D). On the day of cannulation, the lights were turned off in the evening (at sunset; ~1710 h clock time), and remained off until the end of the blood collection period ~45 hours later (see below). The sheep remained in the crates overnight following removal of the catheters at the end of the blood collection period. They were then transferred to undercover outdoor pens for up to 48 hours, and from there to a paddock.

### Light monitoring

The amount of light in the indoor holding room and the undercover outdoor pens was monitored continuously using HOBO temperature/light monitors (Tempcon Instrumentation Ltd., Arundel, UK), with HOBOs positioned throughout the sampling room (n = 4) and undercover pens outside (n = 4). Lux meter readings (in direction of gaze, lux meter held in vertical plane) were collected during blood sampling (daytime; dusk, night, dawn; daytime). Blood sampling in the indoor holding room occurred with lights out. A red headtorch light was used to collect specimens; there were no lights in the corridor, and a blackout curtain hung over the door.

### Placement of jugular cannula

The sheep were fasted overnight prior to the placement of the jugular cannula. Feed was then provided *ad libitum* until the end of that day (i.e. end of the day before blood sampling commenced). From that night onwards and during the 25 hour blood sampling period (14.00 h–15.00 h) food was withheld but water was available *ad libitum*. The sheep were fed immediately on completion of the blood sampling period once the catheter had been removed.

The sheep were conscious throughout the cannula implantation (BD AngioCath, 2.1 × 133 mm). Sheep were administered a local anaesthetic (2–4 mL Lignocaine 20 subcutaneously; Ilium, Australia) after the neck was clipped and sprayed with Povidone Iodine (100 mg/mL Apex Laboratories, Australia). The jugular vein was located and a cannula surgically inserted. The cannula tubing was long enough to be looped around one side of the neck and secured to the back of sheep. A three-way tap was connected to the end of the catheter tubing. All sheep were given a dose of antibiotic (10 mL Rilexine 150, subcutaneously; Virbac, Australia). The sheep were returned to their pens, and when all sheep had recovered, they were fed. Their food was removed when staff left for the day.

### Around-the-clock sample collection

Blood collection commenced at 14:00 h the day following placement of the cannula. The blood samples were collected on the hour (±5 min) hourly for 25 h (blood collection completed at 15:00 h). For blood sample collection via the indwelling cannula, an appropriate dead volume of blood was withdrawn and discarded. Following this, 10 mL of blood was withdrawn into a fresh syringe and transferred to lithium heparin tubes, which were gently inverted 5–8 times. The cannula was flushed with 10 mL of normal saline or heparinised saline (5 IU/mL heparin; Sterisafe, Pfizer, Australia) as required. The samples were centrifuged immediately at 3200 rpm (1620 g) for 10 minutes at 4 °C and split into aliquots. The plasma aliquots were placed immediately in liquid nitrogen or directly into a −80 °C freezer if no liquid nitrogen was available. The samples were stored at −20 °C (for analysis of melatonin and urea nitrogen), or −80 °C (for analysis of LC/MS metabolomics) as specified above, prior to being shipped on dry ice to the UK (University of Surrey) for analysis.

### Sample processing

#### Targeted metabolomic analysis

Two-hourly plasma samples (15.00 h–15.00 h) were measured using the Absolute***IDQ***^®^ p180 targeted metabolomics kit (Biocrates Life Sciences AG, Innsbruck, Austria), and a Waters Xevo TQ-S mass spectrometer coupled to an Acquity UPLC system (Waters Corporation, Milford, MA, USA). Plasma samples (10 μl) were prepared according to the manufacturer’s instructions adding several stable isotope–labelled standards to the samples prior to the derivatisation and extraction steps. Using either LC/MS (liquid chromatography/mass spectrometry) or FIA/MS (flow injection analysis/MS), up to 183 metabolites from 5 different compound classes (namely acylcarnitines, amino acids, biogenic amines, glycerophospholipids and sphingolipids) can be quantified^29^.

Sample order was randomised and 3 levels of quality controls (QC), run on each 96-well plate. The levels of metabolites present in each QC were compared to the expected values and the percent coefficient of variation (CV%) calculated. Data were normalised between batches using the results of quality control level 2 (QC2) repeats across the plate (n = 4) and between plates (n = 5) using Biocrates METIDQ software (QC2 correction). Metabolites where >25% concentrations were below the limit of detection (<LOD) or below lower limit of quantification (≪LLOQ) or above limit of quantification (>LOQ) or blank out of range, or the QC2 coefficient of variance was >30%, were excluded (n = 53). The remaining 130 quantified metabolites comprised 11 acylcarnitines, 20 amino acids, 9 biogenic amines, 76 glycerophospholipids and 14 sphingolipids.

#### Urea measurement

When we saw the pronounced increase in Cit and Arg concentrations, we added urea measurement to our testing protocol. Urea nitrogen measurements were performed on hourly plasma samples across 25 hours using the ADVIA^®^ 1800 Chemistry System (Siemens Healthcare Diagnostics Inc., Camberley, UK), method based on the Roch-Ramel enzymatic reaction using urease and glutamate dehydrogenase. The inter-assay coefficients of variation (CVs) for the plasma urea nitrogen assay were 4.5% at 3.0 mmol/L, 1.5% at 10.2 mmol/L and 1.5% at 19.4 mmol/L (n = 6 at each concentration).

#### Melatonin

Melatonin concentrations were measured on hourly plasma samples by radioimmunoassay as previously described^73^. Melatonin antibody was obtained from Stockgrand Ltd., melatonin measurements were performed by Surrey Assays Ltd (University of Surrey). The limit of detection for the plasma melatonin assay was 3.9 ± 0.8 pg/ml (mean ± SD). The interassay coefficients of variation (CVs) were 11.1% at 13.2 pg/ml, 8.9% at 73.8 pg/ml and 7.2% at 95.3 pg/ml (n = 15 at each concentration). The time of melatonin onset (dim light melatonin onset, DLMO) was calculated using the 25%-threshold method as previously described^74^. In order to control for any inter-animal differences in circadian phase, all the metabolite data were plotted with respect to each animal’s dim light melatonin onset time (DLMO), considered a reliable marker of circadian phase, rather than clock time of day. (In the figures presented, DLMO was annotated as zero; mean (±SEM) was 20.9 ± 0.9 h ≈21.00 h).

### Data analyses

Multivariate analysis was performed by principal component analysis (PCA) and orthogonalized partial least squares discriminant analysis (OPLS-DA), using SIMCA-P v12.0 software (Umetrics, Sweden) and default software settings. The PCA algorithm^75^ transforms the data into a smaller number of dimensions (components) so that the main variation in the data can be visualized in a figure with a smaller number of dimensions. Whereas PCA looks at overall variation (unsupervised), OPLS-DA distinguishes between class-predictive (discriminating) variation (supervised) and non-predictive (orthogonal) variation^76^,^77^. The combined use of these methods allows insight into the extent and underlying patterns of class-predictive variation within the data set as a whole.

Differences in individual metabolite levels were analysed in R version 3.1.2 using the linear models and ANOVA methods in the stats package. Linear models were fitted to the genotype and time of day (13 time points), with the animal as covariate. Significant differences for time of day, genotype, and their interaction were determined using 2-way ANOVA. P-values were corrected for multiple comparisons according to the Benjamini-Hochberg False Discovery Rate (FDR). Metabolites were considered as significant at a FDR cut off <0.05. For testing statistical significance, missing values (<1.2%) were not taken into account.

For data visualisation we used a heat map combined with hierarchical clustering (Euclidean distance and Ward linkage). Quantitative enrichment analysis (QEA) was performed using MetaboAnalyst 3.0 (www.metaboanalyst.ca)^42^. A metabolite concentration (μM) table was inputted, with metabolite names standardised by a built-in tool so that these could be compared with the metabolite set library. The enrichment analysis used a generalised linear model to estimate a Q-statistic (correlation between metabolite concentration and clinical outcomes) for each metabolite set. P-values and corresponding FDR for each metabolic pathway-associated metabolite set that was enriched were calculated.

Prediction models based on combinations of metabolites were made by logistic modelling in R statistical software. First, metabolites were selected that met the following criteria: a significant (FDR <0.05) difference between the control and HD group in the ANOVA model; a (non-cross-validated) Area Under the Curve (AUC) that was significantly higher than for randomly permutated values (p < 0.05, determined by 10,000 permutations); and a difference in plasma concentration of more than 5% between control and HD sheep. For the 37 markers that met these criteria, logistic models were made using log-transformed values. Missing values were imputed as the average value across all samples for that metabolite. To allow for models that would be robust to real-life situations (i.e. sampling can occur at any time point, predictions apply to animals rather than samples), models did not include time as a parameter. The predictive performance of the models was tested by determining the AUC using leave-one-sheep-out cross-validation (LOSOCV), in which a model was trained using data of all time points of all-but-one sheep and tested on the time points of the remaining sheep. Using this approach, models were developed by stepwise testing the result of adding another metabolite to the current model and selecting the metabolite with the highest gain in AUC to be included in the model for the next step. This was repeated until no further improvement in AUC could be achieved. For the eight models obtained (containing one to eight metabolites), predictions were further compared by Receiver Operating Characteristic (ROC) visualisation and determining the predicted sensitivity (detection rate) for a fixed 90% specificity (i.e. 10% false positive rate). In order to determine which time point best discriminates the control and HD groups AUC values for the models were also determined for each time point.

## Additional Information

**How to cite this article**: Skene, D. J. *et al*. Metabolic profiling of presymptomatic Huntington’s disease sheep reveals novel biomarkers. *Sci. Rep.* **7**, 43030; doi: 10.1038/srep43030 (2017).

**Publisher's note:** Springer Nature remains neutral with regard to jurisdictional claims in published maps and institutional affiliations.

## Supplementary Material

Supplementary Figures and Tables

## Figures and Tables

**Table 1:** ANOVA analyses of the metabolite concentrations.

Metabolite	Abbreviation	FDR_genotype	FDR_time	FDR_interaction	Control (μM)mean ± SEM	HD (μM)mean ± SEM
Alanine	Ala	**2.50E-05**	**1.00E-04**	4.20E-01	151.41 ± 2.64	141.89 ± 2.38
Arginine	Arg	**2.40E-08**	**4.10E-07**	1.40E-01	138.68 ± 1.68	149.45 ± 1.95
Asparagine	Asn	**8.80E-05**	**1.10E-17**	4.00E-01	26.28 ± 0.47	24.57 ± 0.43
Citrulline	Cit	**9.50E-24**	**1.30E-04**	6.90E-01	134.79 ± 2.52	161.76 ± 2.37
Glutamine	Gln	**1.90E-02**	9.30E-01	8.00E-01	385.65 ± 4.30	372.94 ± 4.00
Glutamate	Glu	7.80E-01	**8.40E-04**	1.10E-01	40.78 ± 1.35	41.26 ± 1.07
Glycine	Gly	3.90E-01	**5.80E-14**	3.20E-01	1251.40 ± 16.52	1268.56 ± 14.75
Histidine	His	7.10E-01	2.20E-01	6.70E-01	62.39 ± 0.71	61.97 ± 0.91
Isoleucine	Ile	**1.10E-10**	**9.40E-12**	6.20E-01	76.14 ± 1.17	69.68 ± 0.99
Leucine	Leu	**1.30E-16**	**1.70E-09**	5.30E-01	116.18 ± 1.90	102.19 ± 1.73
Lysine	Lys	5.60E-01	**5.20E-11**	3.20E-01	128.39 ± 3.24	125.95 ± 3.69
Methionine	Met	**1.30E-06**	**4.00E-10**	3.90E-01	20.94 ± 0.39	19.47 ± 0.36
Ornithine	Orn	9.50E-01	**4.10E-02**	7.10E-01	84.07 ± 1.57	84.19 ± 1.50
Phenylalanine	Phe	**2.30E-03**	1.30E-01	2.90E-01	57.71 ± 0.69	55.31 ± 0.70
Proline	Pro	8.20E-01	**1.20E-09**	2.40E-01	75.45 ± 0.98	75.75 ± 0.93
Serine	Ser	8.10E-01	**1.30E-09**	8.00E-01	126.54 ± 1.67	125.95 ± 1.77
Threonine	Thr	**2.30E-10**	**2.20E-11**	5.20E-01	160.23 ± 2.38	144.65 ± 4.35
Tryptophan	Trp	8.00E-01	**4.00E-33**	4.40E-01	36.44 ± 0.81	36.66 ± 0.76
Tyrosine	Tyr	**1.30E-06**	**7.20E-07**	4.20E-01	63.06 ± 1.01	58.90 ± 0.91
Valine	Val	**5.80E-20**	**1.60E-05**	7.40E-01	164.49 ± 2.69	145.39 ± 2.48
Assymetric dimethylarginine	ADMA	**1.40E-03**	**1.80E-02**	**2.80E-02**	2.26 ± 0.03	2.38 ± 0.03
alpha-Aminoadipic acid	alpha-AAA	**4.70E-02**	**1.30E-13**	5.60E-01	5.03 ± 0.14	5.28 ± 0.18
Carnosine		**3.10E-10**	9.30E-01	6.90E-01	13.83 ± 0.28	15.12 ± 0.22
Creatinine		3.80E-01	4.90E-01	4.40E-01	179.43 ± 12.55	189.81 ± 10.34
Kynurenine		**1.70E-04**	**5.10E-23**	7.90E-01	2.37 ± 0.10	2.58 ± 0.07
Sarcosine		**5.60E-20**	**6.70E-22**	5.10E-01	4.70 ± 0.09	4.10 ± 0.10
Serotonin		1.10E-01	6.60E-02	2.30E-01	6.68 ± 0.31	6.00 ± 0.25
Trans-4-hydroxyproline	t4-OH-Pro	**1.00E-11**	**2.30E-16**	4.00E-01	21.60 ± 0.36	23.63 ± 0.43
Taurine		2.60E-01	**2.30E-08**	4.90E-01	29.95 ± 1.22	31.25 ± 1.12
Symmetric dimethylarginine	SDMA	**2.80E-02**	**3.30E-24**	5.40E-02	1.42 ± 0.03	1.47 ± 0.03
Carnitine	C0	7.40E-01	**1.10E-06**	8.40E-01	44.64 ± 1.08	45.07 ± 1.00
Tetradecenoylcarnitine	C14:1	**1.60E-12**	**2.70E-10**	4.70E-01	0.03 ± 0.00	0.02 ± 0.00
Hydroxytetradecenoylcarnitine	C14:1-OH	**1.30E-03**	**2.10E-20**	8.40E-01	0.03 ± 0.00	0.02 ± 0.00
Hexadecenoylcarnitine	C16:1	**1.00E-18**	**1.00E-17**	4.90E-01	0.02 ± 0.00	0.02 ± 0.00
Hydroxyhexadecenoylcarnitine	C16:1-OH	**1.20E-08**	**1.20E-24**	8.00E-01	0.03 ± 0.00	0.03 ± 0.00
Octadecenoylcarnitine	C18:1	**1.20E-18**	**6.80E-23**	5.80E-01	0.08 ± 0.00	0.07 ± 0.00
Acetylcarnitine	C2	8.50E-01	**4.30E-32**	6.30E-01	30.26 ± 0.73	30.40 ± 0.80
Malonylhydroxybutyrylcarnitine	C3-DC (C4-OH)	**2.00E-08**	**4.90E-09**	8.30E-01	0.09 ± 0.00	0.10 ± 0.00
Hydroxyvalerylcarnitine	C5-OH (C3-DC-M)	**5.10E-03**	**7.70E-03**	6.50E-01	0.08 ± 0.00	0.08 ± 0.00
Glutaconylcarnitine	C5:1-DC	**4.30E-02**	**1.40E-04**	7.40E-01	0.02 ± 0.00	0.02 ± 0.00
Glutarylcarnitine	C5-DC (C6-OH)	1.50E-01	9.90E-01	2.70E-01	0.02 ± 0.00	0.02 ± 0.00
lysoPC a C16:0		4.90E-01	2.70E-01	2.60E-01	28.10 ± 0.34	27.73 ± 0.37
lysoPC a C16:1		**8.00E-04**	**2.60E-04**	7.10E-01	1.63 ± 0.03	1.54 ± 0.02
lysoPC a C17:0		**1.40E-14**	**1.20E-02**	8.00E-01	2.92 ± 0.08	3.34 ± 0.06
lysoPC a C18:0		7.40E-02	**5.50E-05**	3.80E-01	18.69 ± 0.32	19.30 ± 0.28
lysoPC a C18:1		9.90E-02	6.50E-01	5.70E-01	24.55 ± 0.38	23.77 ± 0.39
lysoPC a C18:2		4.70E-01	**5.70E-03**	5.80E-01	18.40 ± 0.39	18.10 ± 0.30
lysoPC a C20:3		6.50E-02	**7.90E-05**	9.80E-01	0.81 ± 0.02	0.84 ± 0.01
lysoPC a C20:4		8.70E-01	**2.40E-02**	6.40E-01	4.52 ± 0.09	4.53 ± 0.07
lysoPC a C24:0		**4.10E-02**	**1.90E-03**	6.90E-01	0.14 ± 0.00	0.15 ± 0.00
lysoPC a C26:0		7.90E-01	**5.80E-03**	9.90E-01	0.11 ± 0.00	0.11 ± 0.00
lysoPC a C26:1		**7.20E-08**	8.00E-02	7.10E-01	0.07 ± 0.00	0.08 ± 0.00
lysoPC a C28:0		5.10E-01	3.50E-01	1.00E + 00	0.20 ± 0.00	0.19 ± 0.00
lysoPC a C28:1		**4.90E-11**	9.00E-01	9.30E-01	0.48 ± 0.01	0.57 ± 0.02
PC aa C28:1		**5.60E-15**	4.20E-01	4.20E-01	0.32 ± 0.01	0.29 ± 0.00
PC aa C30:0		**8.40E-14**	3.40E-01	6.00E-01	1.15 ± 0.03	1.02 ± 0.02
PC aa C32:0		**2.80E-08**	**4.50E-02**	7.90E-02	4.95 ± 0.12	4.37 ± 0.08
PC aa C32:1		**1.20E-03**	**1.30E-02**	9.10E-01	1.80 ± 0.05	1.67 ± 0.03
PC aa C32:3		**1.40E-04**	**4.00E-05**	5.20E-01	0.22 ± 0.00	0.21 ± 0.00
PC aa C34:1		**1.80E-18**	3.60E-01	3.80E-01	73.98 ± 1.84	65.40 ± 1.31
PC aa C34:2		**2.60E-05**	**7.40E-09**	5.20E-01	82.67 ± 3.27	77.28 ± 2.72
PC aa C34:3		**2.00E-04**	**1.20E-23**	8.00E-01	9.75 ± 0.23	10.37 ± 0.18
PC aa C34:4		8.20E-02	**2.10E-05**	3.20E-01	0.33 ± 0.01	0.34 ± 0.01
PC aa C36:0		**5.30E-06**	**1.70E-02**	8.00E-01	4.41 ± 0.13	4.07 ± 0.07
PC aa C36:1		**5.90E-05**	8.00E-02	4.10E-01	55.83 ± 0.95	52.70 ± 0.96
PC aa C36:2		**3.60E-02**	**8.60E-06**	4.30E-01	83.68 ± 2.29	81.10 ± 1.78
PC aa C36:3		2.00E-01	**1.40E-17**	7.10E-01	27.83 ± 0.60	28.50 ± 0.45
PC aa C36:4		7.10E-01	**1.30E-08**	4.00E-01	16.19 ± 0.43	16.05 ± 0.35
PC aa C36:5		**4.00E-14**	**3.10E-18**	5.90E-02	3.26 ± 0.08	3.71 ± 0.08
PC aa C36:6		**1.10E-02**	2.90E-01	4.70E-01	0.17 ± 0.01	0.18 ± 0.00
PC aa C38:0		**1.90E-02**	2.00E-01	3.90E-01	1.05 ± 0.03	1.01 ± 0.01
PC aa C38:3		**1.90E-02**	5.20E-01	6.80E-01	7.81 ± 0.18	7.49 ± 0.16
PC aa C38:4		**8.50E-04**	**8.40E-04**	5.20E-01	30.98 ± 0.69	29.49 ± 0.47
PC aa C38:5		7.20E-02	5.10E-02	3.40E-01	22.48 ± 0.45	23.18 ± 0.45
PC aa C38:6		**5.00E-04**	**1.50E-04**	4.40E-01	8.90 ± 0.25	9.48 ± 0.18
PC aa C40:2		7.90E-01	**3.90E-03**	5.50E-01	0.07 ± 0.00	0.07 ± 0.00
PC aa C40:3		**9.20E-04**	8.40E-01	3.20E-01	0.40 ± 0.01	0.37 ± 0.01
PC aa C40:4		**2.00E-11**	7.90E-01	7.10E-01	3.18 ± 0.07	2.85 ± 0.06
PC aa C40:5		**3.30E-03**	**6.10E-03**	3.80E-01	18.40 ± 0.39	17.53 ± 0.41
PC aa C40:6		**5.20E-04**	**1.80E-03**	5.20E-01	17.41 ± 0.49	18.45 ± 0.32
PC aa C42:1		1.90E-01	9.50E-01	5.30E-01	0.06 ± 0.00	0.06 ± 0.00
PC aa C42:4		9.50E-02	3.40E-01	6.30E-01	0.07 ± 0.00	0.07 ± 0.00
PC aa C42:5		**6.10E-03**	**2.70E-03**	3.90E-01	0.49 ± 0.01	0.47 ± 0.01
PC aa C42:6		**4.40E-08**	**1.40E-04**	3.90E-01	0.79 ± 0.03	0.86 ± 0.02
PC ae C30:0		**8.40E-04**	**3.30E-02**	8.00E-01	0.29 ± 0.01	0.27 ± 0.00
PC ae C30:2		**3.30E-13**	4.40E-01	5.60E-01	0.16 ± 0.00	0.18 ± 0.00
PC ae C32:1		**2.00E-05**	5.10E-01	8.60E-01	0.65 ± 0.02	0.56 ± 0.01
PC ae C32:2		**1.50E-04**	2.00E-01	5.10E-01	0.48 ± 0.01	0.46 ± 0.01
PC ae C34:0		8.80E-01	7.90E-02	4.10E-01	2.85 ± 0.10	2.83 ± 0.05
PC ae C34:1		**5.90E-06**	**2.90E-02**	4.90E-01	5.44 ± 0.14	5.03 ± 0.08
PC ae C34:2		**6.20E-12**	**9.70E-08**	5.20E-01	5.19 ± 0.11	4.70 ± 0.08
PC ae C34:3		**2.20E-17**	**5.00E-07**	2.90E-01	2.10 ± 0.04	1.87 ± 0.04
PC ae C36:0		7.60E-01	**2.20E-04**	6.90E-01	1.66 ± 0.06	1.67 ± 0.03
PC ae C36:1		**1.60E-03**	**2.10E-03**	8.00E-01	14.65 ± 0.47	13.92 ± 0.25
PC ae C36:2		4.90E-01	**3.90E-16**	9.50E-01	8.69 ± 0.23	8.80 ± 0.14
PC ae C36:3		5.20E-01	**2.20E-14**	6.70E-01	3.00 ± 0.08	2.96 ± 0.05
PC ae C36:4		**7.90E-06**	3.40E-01	2.00E-01	1.49 ± 0.03	1.39 ± 0.02
PC ae C36:5		**5.00E-14**	1.40E-01	3.00E-01	1.42 ± 0.03	1.27 ± 0.02
PC ae C38:0		1.20E-01	6.20E-01	3.90E-01	0.72 ± 0.02	0.74 ± 0.01
PC ae C38:1		****2.60E-44****	1.10E-01	****9.20E-03****	17.19 ± 2.55	7.94 ± 1.18
PC ae C38:2		****2.40E-03****	**3.00E-11**	7.10E-01	1.57 ± 0.07	1.48 ± 0.03
PC ae C38:3		8.10E-01	**1.50E-07**	9.20E-01	1.80 ± 0.06	1.81 ± 0.03
PC ae C38:4		3.00E-01	3.10E-01	3.90E-01	2.63 ± 0.07	2.58 ± 0.04
PC ae C38:5		3.60E-01	7.30E-01	1.50E-01	1.99 ± 0.04	2.03 ± 0.04
PC ae C38:6		**1.90E-02**	5.80E-01	3.20E-01	1.28 ± 0.03	1.23 ± 0.02
PC ae C40:1		**3.00E-07**	**2.10E-02**	5.10E-01	0.22 ± 0.01	0.26 ± 0.01
PC ae C40:2		**3.20E-04**	6.30E-01	8.60E-01	0.59 ± 0.01	0.56 ± 0.01
PC ae C40:3		**4.10E-03**	5.80E-01	9.80E-01	0.55 ± 0.02	0.52 ± 0.01
PC ae C40:4		**6.00E-03**	8.90E-01	8.20E-01	1.05 ± 0.03	1.01 ± 0.02
PC ae C40:5		7.50E-02	8.90E-01	7.60E-01	2.26 ± 0.06	2.34 ± 0.05
PC ae C40:6		**5.30E-06**	8.60E-01	4.70E-01	1.69 ± 0.05	1.81 ± 0.03
PC ae C42:1		5.10E-01	**1.90E-02**	6.50E-01	0.09 ± 0.00	0.09 ± 0.00
PC ae C42:2		**1.90E-02**	4.20E-01	8.10E-01	0.13 ± 0.00	0.12 ± 0.00
PC ae C42:3		**8.50E-05**	**1.30E-07**	3.80E-01	0.13 ± 0.00	0.14 ± 0.00
PC ae C42:4		**6.70E-03**	7.80E-01	1.90E-01	0.07 ± 0.00	0.06 ± 0.00
PC ae C44:6		5.80E-01	4.90E-01	5.80E-01	0.05 ± 0.00	0.05 ± 0.00
SM (OH) C14:1		**1.40E-09**	8.30E-01	5.00E-01	7.39 ± 0.13	6.74 ± 0.10
SM (OH) C16:1		**5.90E-05**	8.70E-01	4.20E-01	11.60 ± 0.24	10.88 ± 0.18
SM (OH) C22:1		**6.00E-08**	8.40E-01	3.20E-01	27.60 ± 0.54	25.07 ± 0.47
SM (OH) C22:2		**3.50E-07**	8.00E-01	5.40E-01	16.40 ± 0.34	15.01 ± 0.26
SM (OH) C24:1		**7.60E-08**	6.50E-01	5.40E-01	1.73 ± 0.04	1.56 ± 0.03
SM C16:0		**1.10E-17**	5.20E-01	3.40E-01	94.90 ± 1.61	82.17 ± 1.35
SM C16:1		**2.50E-12**	7.10E-01	3.20E-01	5.51 ± 0.10	4.93 ± 0.10
SM C18:0		**4.80E-13**	2.00E-01	2.40E-01	19.51 ± 0.46	17.21 ± 0.29
SM C18:1		**1.20E-09**	8.30E-01	1.70E-01	5.92 ± 0.14	5.35 ± 0.09
SM C20:2		6.60E-01	9.80E-01	9.30E-01	0.13 ± 0.00	0.13 ± 0.00
SM C24:0		**1.20E-09**	8.00E-01	2.70E-01	15.38 ± 0.33	13.73 ± 0.24
SM C24:1		**1.90E-09**	4.30E-01	3.80E-01	33.71 ± 0.82	29.89 ± 0.58
SM C26:0		**4.30E-03**	8.90E-01	5.00E-01	0.32 ± 0.01	0.30 ± 0.01
SM C26:1		**5.20E-04**	6.90E-01	4.00E-01	0.24 ± 0.01	0.21 ± 0.01

Individual metabolite levels were analysed in R version 3.1.2 using the linear models and ANOVA methods. Linear models were fitted to the genotype and time of day, with the animal as covariate. Significant differences for time of day, genotype, and their interaction were determined using 2-way ANOVA. P-values were corrected for multiple comparisons according to the Benjamini-Hochberg False Discovery Rate (FDR). Metabolites were considered as significant at a FDR cut off <0.05 (highlighted in bold). 24 h mean values (±SEM), derived from all the time points, for both the control and HD sheep are also shown.
