# Supplementary material for: Metabolic profiling of presymptomatic Huntington’s disease sheep reveals novel biomarkers
Source: Sci Rep. 2017 Feb 22;7:43030. doi: 10.1038/srep43030 (PMC5320451; doi:10.1038/srep43030)
Supplement: Supplementary Figures and Tables [file srep43030-s1.pdf]

Metabolic profiling of presymptomatic Huntington's disease sheep reveals novel biomarkers

Debra J. Skene[1*], Benita Middleton[1], Cara K. Fraser[2], Jeroen L.A. Pennings[3], Timothy R. Kuchel[2], Skye Rudiger[4], C. Simon Bawden[4], A. Jennifer Morton[5]*

[1]Chronobiology, Faculty of Health and Medical Sciences, University of Surrey, Guildford GU2 7XH, United Kingdom; [2]Preclinical, Imaging & Research Laboratories (PIRL), SAHMRI, Gilles Plains, Adelaide, Australia; [3]National Institute for Public Health and the Environment (RIVM), 3720 BA Bilthoven, The Netherlands; [4]South Australian Research and Development Institute, Roseworthy, South Australia; [5]Department of Physiology, Development and Neuroscience, University of Cambridge, Downing Street, Cambridge CB2 3DY, United Kingdom.

*Authors for Correspondence

Professor Jenny Morton: ajm41@cam.ac.uk

Professor Debra J. Skene: d.skene@surrey.ac.uk

Key words: OVT73, wasting, urea, urea cycle, nitric oxide, branched chain amino acids, kynurenine, kynurenic acid, sphingolipids, sleep, circadian, neurodegeneration

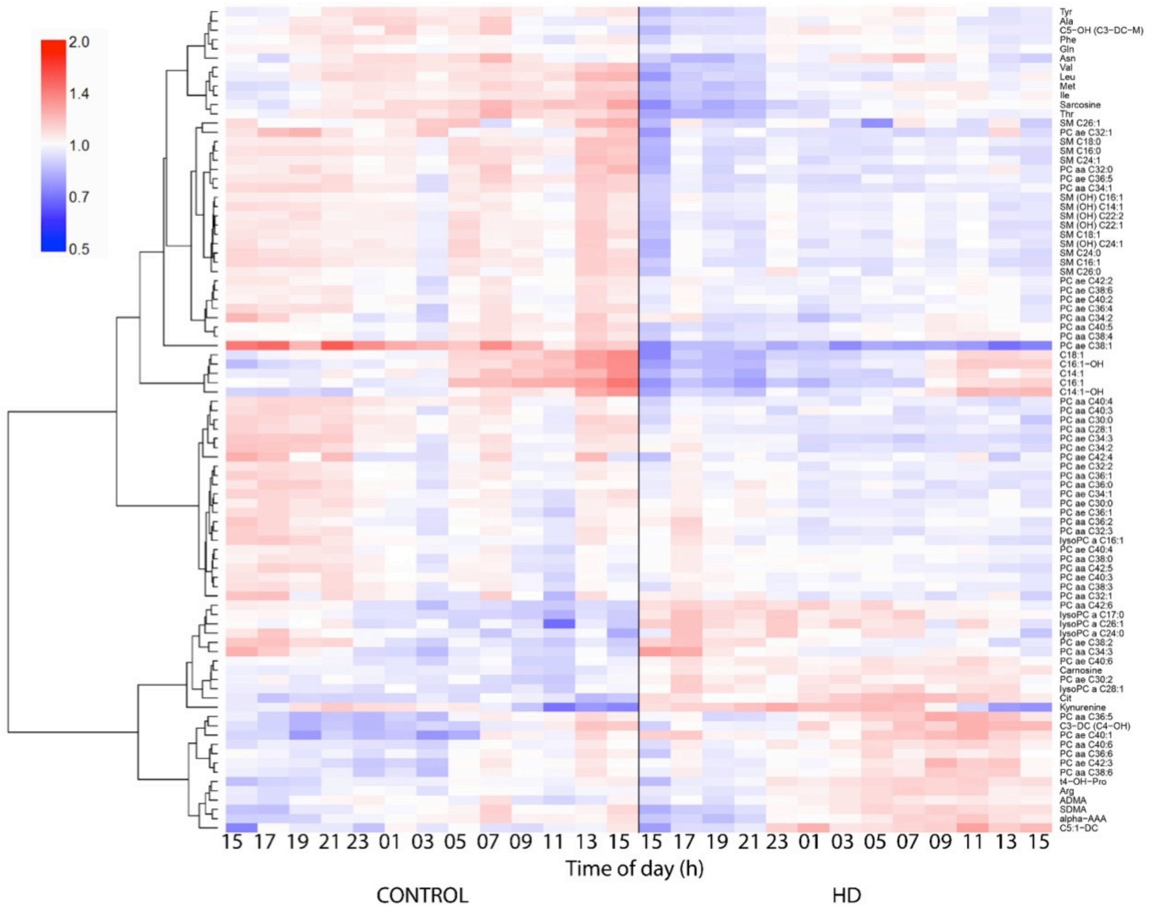

Supplementary Figure 1 Heat map combined with hierarchical clustering (Euclidean distance and Ward linkage) of 89 metabolites showing significant changes between genotypes. Metabolites were measured across a 24 h period (15:00 - 15:00 h) in both the control and HD sheep. Colour values are based on the relative value compared to the average value of each metabolite in the complete data set. Higher levels of metabolites are shown in red, lower levels are shown in blue.

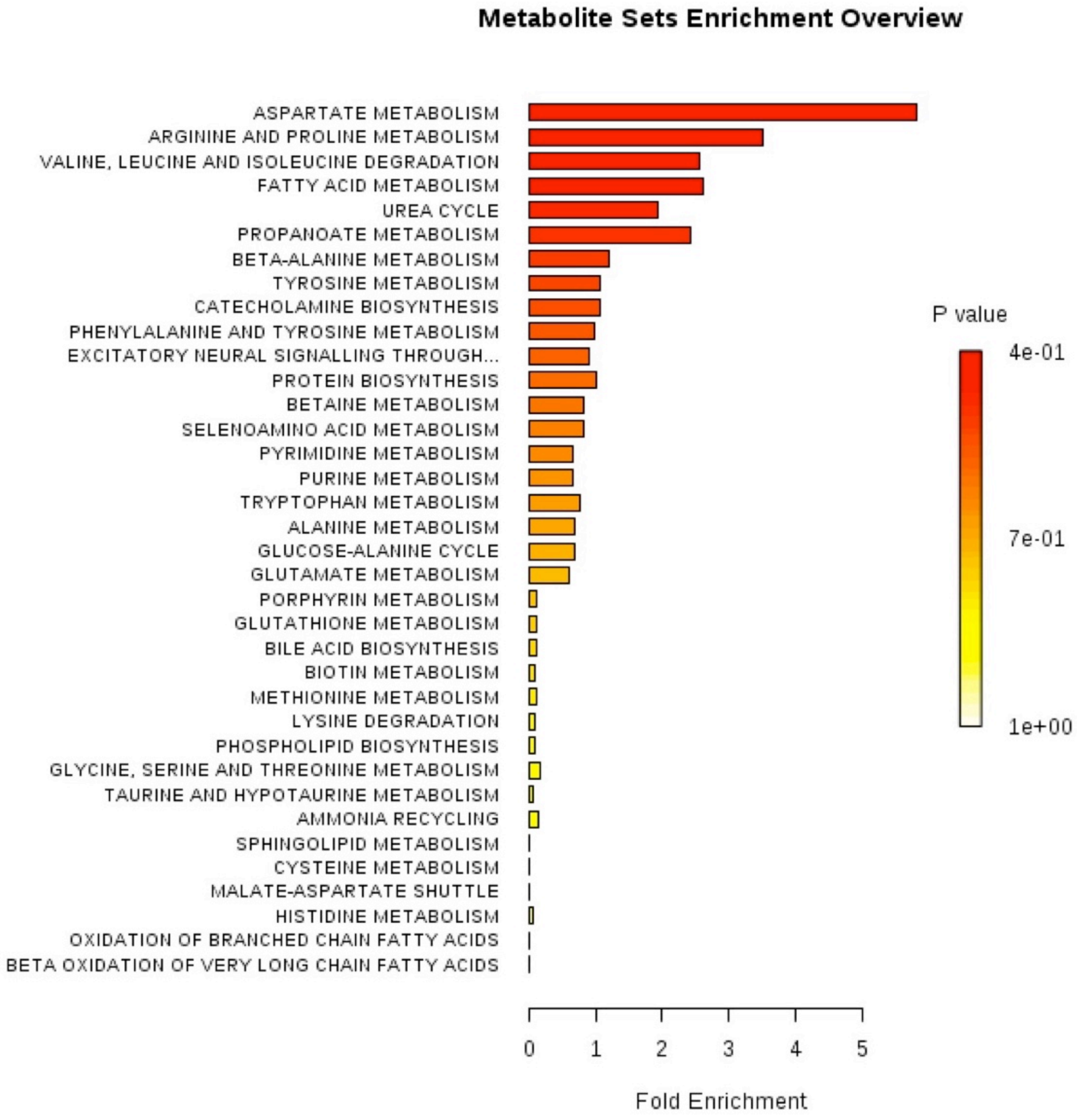

Supplementary Figure 2 Summary plot for quantitative enrichment analysis performed using MetaboAnalyst 3.0. An overview of the metabolite sets enriched is presented.

Supplement Table S1: p(corr) values of the measured metabolites in OPLS-DA models comparing control and HD sheep.

Metabolite	p(corr)	Metabolite	p(corr)	Metabolite
SM C16:0	-0.353	PC aa C40:3	-0.121	PC ae C40:5
Leucine	-0.338	PC aa C38:4	-0.121	Glutaconylcarnitine
Hexadecenoylcarnitine	-0.316	PC ae C40:2	-0.119	Creatinine
Valine	-0.314	PC ae C42:4	-0.111	Taurine
Tetradecenoylcarnitine	-0.307	Glutarylcarnitine	-0.108	PC ae C38:0
SM C18:0	-0.272	Glutamine	-0.108	PC aa C34:4
PC aa C30:0	-0.270	PC ae C38:6	-0.091	Glycine
SM C24:0	-0.265	PC aa C40:5	-0.091	Glutamate
SM C16:1	-0.264	PC aa C38:3	-0.091	PC aa C38:5
PC aa C28:1	-0.262	Hydroxytetradecenoylcarnitine	-0.090	Symmetric dimethylarginine
Octadecenoylcarnitine	-0.261	PC aa C36:1	-0.089	lysoPC a C20:3
Sarcosine	-0.257	PC aa C42:4	-0.086	lysoPC a C18:0
PC ae C36:5	-0.255	PC ae C42:2	-0.084	PC aa C36:6
Isoleucine	-0.254	PC ae C40:4	-0.080	PC aa C36:3
SM (OH) C14:1	-0.245	PC ae C40:3	-0.079	alpha-aminodipic acid
SM C24:1	-0.243	PC aa C42:5	-0.079	PC aa C42:1
PC ae C34:3	-0.242	PC aa C34:2	-0.076	PC aa C40:6
PC aa C32:0	-0.240	PC ae C38:2	-0.062	lysoPC a C24:0
SM C18:1	-0.233	lysoPC a C28:0	-0.060	PC aa C38:6
SM (OH) C24:1	-0.227	lysoPC a C18:1	-0.060	PC ae C40:6
SM (OH) C22:1	-0.220	PC ae C36:1	-0.058	Asymmetric dimethylarginine
PC ae C38:1	-0.214	PC aa C38:0	-0.058	PC ae C42:3
PC ae C34:2	-0.207	PC aa C36:2	-0.057	Kynurenine
SM C26:1	-0.206	lysoPC a C16:0	-0.046	PC aa C42:6
PC ae C32:1	-0.203	lysoPC a C18:2	-0.046	PC aa C34:3
PC aa C40:4	-0.200	SM C20:2	-0.040	Malonylhydroxybutyrylcarnitine
PC aa C34:1	-0.197	PC ae C38:4	-0.033	trans-4-hydroxyproline
SM (OH) C22:2	-0.194	Lysine	-0.022	lysoPC a C28:1
Tyrosine	-0.189	lysoPC a C26:0	-0.022	PC ae C30:2
Threonine	-0.177	PC ae C36:3	-0.019	PC ae C40:1
Hydroxyhexadecenoylcarnitine	-0.175	Acetylcarnitine	-0.012	Carnosine
PC ae C36:4	-0.172	lysoPC a C20:4	-0.010	lysoPC a C17:0
Hydroxyvalerylcarnitine	-0.170	Histidine	0.001	PC aa C36:5
Methionine	-0.161	Tryptophan	0.006	lysoPC a C26:1
SM (OH) C16:1	-0.160	PC ae C34:0	0.006	Arginine
Alanine	-0.157	PC aa C36:4	0.007	Citrulline

**PC aa C32:3**	-0.150	**Serine**	0.014
**lysoPC a C16:1**	-0.148	**PC ae C38:3**	0.017
**PC ae C34:1**	-0.148	**Proline**	0.019
**Phenylalanine**	-0.147	**PC ae C44:6**	0.023
**PC ae C32:2**	-0.143	**Carnitine**	0.025
**Asparagine**	-0.138	**PC ae C38:5**	0.033
**PC ae C30:0**	-0.134	**PC ae C36:0**	0.034
**PC aa C32:1**	-0.132	**Ornithine**	0.036
**PC aa C36:0**	-0.131	**PC aa C40:2**	0.039
**SM C26:0**	-0.122	**PC ae C36:2**	0.042
**Serotonin**	-0.122	**PC ae C42:1**	0.045

The rank order of the metabolites shown in Table S1 is also depicted in Figure 1c from left to right. The metabolites are colour coded according to metabolite class as follows: amino acids and biogenic amines (blue); acylcarnitines (green); lysophosphatidylcholine acyl (lyso PC a) (dark orange); phosphatidylcholine diacyl (PC aa) (yellow); phosphatidylcholine acyl-akyl (PC ae) (light orange); sphingolipids (SM) (brown).

Supplement Table S2: Comparison of the models shown in Figure 7.

Model	Number of metabolites	Metabolites used	AUC (95% CI)	Sensitivity at 90% specificity (%) (95% CI)
Model_1	1	citrulline	0.664 (0.602-0.726)	17.7 (3.9 - 43.1)
Model_2	2	citrulline + valine	0.702 (0.643-0.760)	36.5 (18.8 - 51.4)
Model_3	3	above + PC aa C40:4	0.746 (0.691-0.802)	50.8 (38.1 - 63)
Model_4	4	above + PC aa C36:5	0.775 (0.723-0.827)	47.0 (34.3 - 60.2)
Model_5	5	above + lysoPC a C17:0	0.813 (0.767-0.859)	56.6 (47.5 - 66.3)
Model_6	6	above + SM (OH) C24:1	0.878 (0.841-0.915)	68.5 (57.5 - 76.3)
Model_7	7	above + threonine	0.935 (0.910-0.960)	81.2 (73.5 - 86.7)
Model_8	8	above + C14:1	0.938 (0.913-0.963)	80.1 (71.3 - 88.4)
